# Supplementary material for: The Petasites hybridus CO2 Extract (Ze 339) Blocks SARS-CoV-2 Replication In Vitro
Source: Viruses. 2022 Jan 7;14(1):106. doi: 10.3390/v14010106 (PMC8781559; doi:10.3390/v14010106)
Supplement: Supplementary file 1 [file viruses-14-00106-s001.zip › viruses-1514885-supplementary.pdf]

Supplemental material to:

The *Petasites hybridus* CO_2-extract (Ze 339) blocks SARS-CoV-2 replication *in vitro*

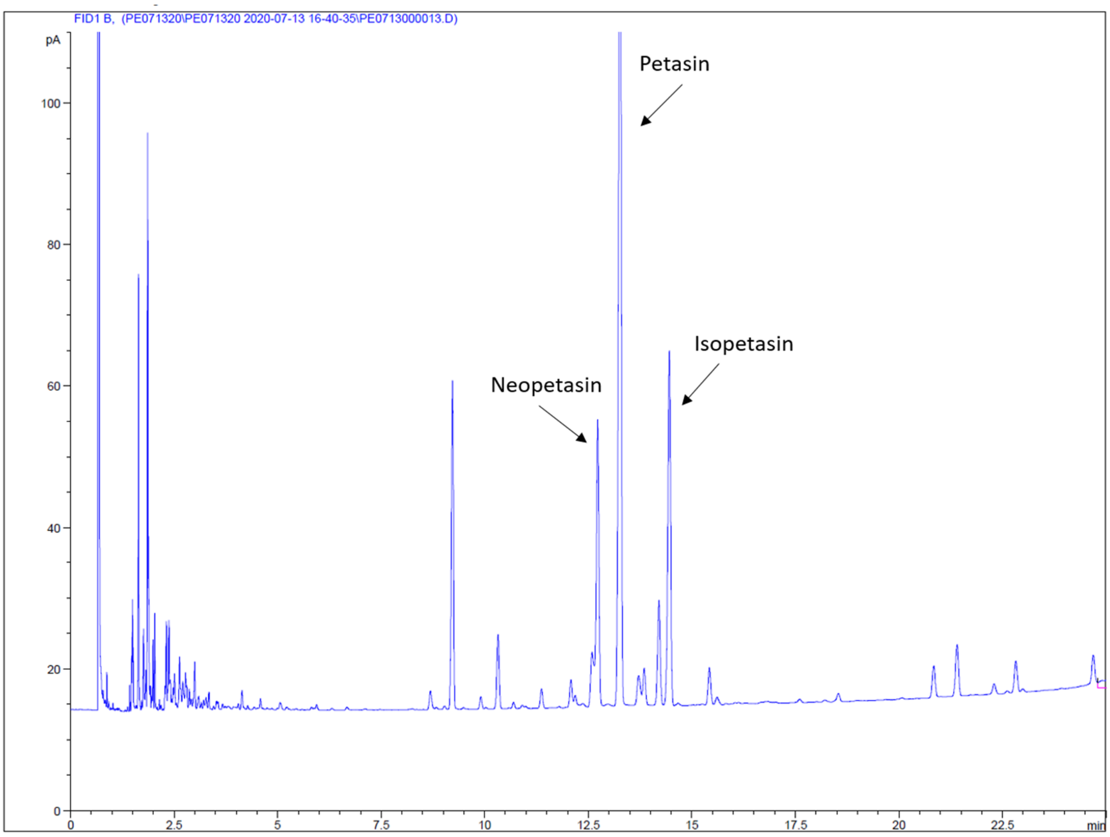

Figure S1. Gas chromatogram of *Petasites hybridus* leaf extract Ze 339 (batch 150056). Quantitative determination of total petasins (petasin, isopetasin, neopetasin), the active compounds of Ze 339 using gas chromatography and a flame ionization detector (FID). GC-column 100% polydimethylsiloxane (e.g. DB-1, length: 25 m, ID: 0.32 mm, dF: 0.52 µm); Injector temperature: 270 °C; Injection volume 1 µl.

(a)

drugdiscovery.utep.edu/redial/index2.php

Back

RESULTS

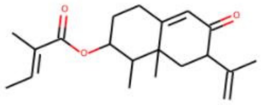

LogP (Log units)	LogS (Log units)	Molecular Wt. (g/mol)	Formula
5.17	-4.37	316.44	C20H28O3

External reference:

PubChem CID	Drug Central ID
3504628	Not Found

Synonyms: -|-

Processed SMILES string:

C=C(C)C1CC2(C)C(=CC1=O)CCC(OC(=O)C(C)=CC)C2C

Prediction Results

	Class	Prediction	Confidence
Live Virus Infectivity	SARS-CoV-2 cytopathic effect (CPE)	INACTIVE	0.93
	SARS-CoV-2 cytopathic effect (host tox Counter) / Cytotoxicity	INACTIVE	0.78
Viral Entry	Spike-ACE2 protein-protein interaction (AlphaLISA)	ACTIVE	0.63
	Spike-ACE2 protein-protein interaction (TruHit Counter)	ACTIVE	0.73
	ACE2 enzymatic activity	INACTIVE	0.84
Viral Replication	3CL enzymatic activity	ACTIVE	0.53
In vitro Infectivity	SARS-CoV pseudotyped particle entry (CoV-PPE)	INACTIVE	0.56
	SARS-CoV pseudotyped particle entry counter screen (CoV-PPE_cs)	INACTIVE	0.62
	MERS-CoV pseudotyped particle entry (MERS-PPE)	ACTIVE	0.69
	MERS-CoV pseudotyped particle entry counter screen (MERS-PPE_cs)	INACTIVE	0.67
Human Cell Toxicity	Human fibroblast toxicity (hCYTOX)	INACTIVE	0.74
Host Protein	Sigma1 Receptor (sigma1R)	INACTIVE	0.81

Promising drugs are those that:

- **Are active in CPE and are inactive in cytotox**

AND

- **Are inactive in ACE2**

AND

- **Are active in 3CL**

AND/OR

- **Are active in at least one of the following: AlphaLISA, CoV-PPE, MERS-PPE. While they are inactive in the counter screen, respectively: TruHit, CoV-PPE_cs, MERS-PPE_cs**

AND

- **Are inactive in hCYTOX**

(**b**)

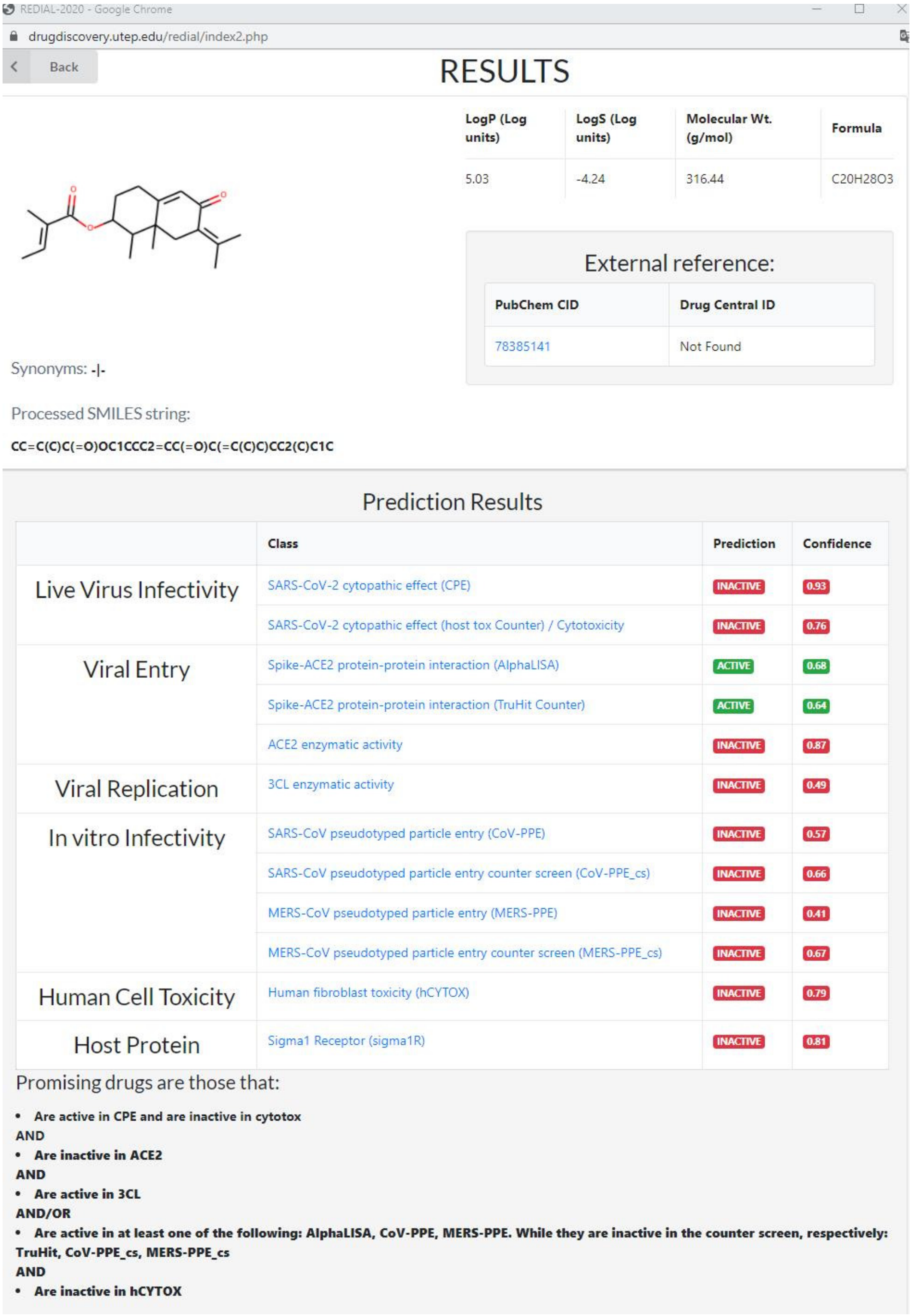

REDIAL-2020 - Google Chrome

drugdiscovery.utep.edu/redial/index2.php

Back

RESULTS

LogP (Log units)	LogS (Log units)	Molecular Wt. (g/mol)	Formula
5.03	-4.24	316.44	C20H28O3

External reference:

PubChem CID	Drug Central ID
78385141	Not Found

Synonyms: -|-

Processed SMILES string:

CC=C(C)C(=O)OC1CCC2=CC(=O)C(=C(C)C)CC2(C)C1C

Prediction Results

	Class	Prediction	Confidence
Live Virus Infectivity	SARS-CoV-2 cytopathic effect (CPE)	INACTIVE	0.93
	SARS-CoV-2 cytopathic effect (host tox Counter) / Cytotoxicity	INACTIVE	0.76
Viral Entry	Spike-ACE2 protein-protein interaction (AlphaLISA)	ACTIVE	0.68
	Spike-ACE2 protein-protein interaction (TruHit Counter)	ACTIVE	0.64
	ACE2 enzymatic activity	INACTIVE	0.87
Viral Replication	3CL enzymatic activity	INACTIVE	0.49
In vitro Infectivity	SARS-CoV pseudotyped particle entry (CoV-PPE)	INACTIVE	0.57
	SARS-CoV pseudotyped particle entry counter screen (CoV-PPE_cs)	INACTIVE	0.66
	MERS-CoV pseudotyped particle entry (MERS-PPE)	INACTIVE	0.41
	MERS-CoV pseudotyped particle entry counter screen (MERS-PPE_cs)	INACTIVE	0.67
Human Cell Toxicity	Human fibroblast toxicity (hCYTOX)	INACTIVE	0.79
Host Protein	Sigma1 Receptor (sigma1R)	INACTIVE	0.81

Promising drugs are those that:

- **Are active in CPE and are inactive in cytotox**

AND

- **Are inactive in ACE2**

AND

- **Are active in 3CL**

AND/OR

- **Are active in at least one of the following: AlphaLISA, CoV-PPE, MERS-PPE. While they are inactive in the counter screen, respectively: TruHit, CoV-PPE_cs, MERS-PPE_cs**

AND

- **Are inactive in hCYTOX**

Figure S2. Prediction of anti-viral mechanisms (**a**) Petasin (**b**) Isopetasin (http://drugcentral.org/Redial)

Table S1. Druglikeness of petasin, isopetasin and neopetasin (SwissADME: a free web tool to evaluate pharmacokinetics, drug-likeness and medicinal chemistry friendliness of small molecules [1]).

	Criterion	Petasin	Neopetasin	Isopetasin	Acceptance
Lipinski [2]	MW ≤ 500	316.4	316.4	316.4	yes
	MLogP ≤ 4.15	3.48	3.48	3.48	yes
	H-Bond Donors ≤ 5	0	0	0	yes
	H-Bond Acceptors ≤ 10	3	3	3	yes
Ghose [3]	150 ≤ MW ≤ 480	316.4	316.4	316.4	yes
	-0.4 ≤ WLogP ≤ 5.6	4.39	4.39	4.45	yes
	40 ≤ MR ≤ 130	93.8	93.8	93.8	yes
	20 ≤ atoms ≤ 70	51	51	51	yes
Veber [4]	Rotatable bonds ≤ 10	4	4	3	yes
	TPSA ≤ 140 Å^2	43.37	43.37	43.37	yes

MW = molecular weight, MR = molecular refractivity, MLogP = rule-based Moriguchi Log P [2], WLogP = a purely atomistic method based Log P estimate on the fragmental system ([5], TPSA = Topological polar surface area.

References

1. Kc, G.B.; Bocci, G.; Verma, S.; Hassan, M.M.; Holmes, J.; Yang, J.J.; Sirimulla, S.; Oprea, T.I. A machine learning platform to estimate anti-SARS-CoV-2 activities. *Nature Machine Intelligence* **2021**, *3*, 527-535, doi:10.1038/s42256-021-00335-w.
2. Daina, A.; Michielin, O.; Zoete, V. SwissADME: a free web tool to evaluate pharmacokinetics, drug-likeness and medicinal chemistry friendliness of small molecules. *Sci Rep* **2017**, *7*, 42717, doi:10.1038/srep42717.
3. Lipinski, C.A.; Lombardo, F.; Dominy, B.W.; Feeney, P.J. Experimental and computational approaches to estimate solubility and permeability in drug discovery and development settings. *Adv Drug Deliv Rev* **2001**, *46*, 3-26, doi:10.1016/s0169-409x(00)00129-0.
4. Ghose, A.K.; Viswanadhan, V.N.; Wendoloski, J.J. A knowledge-based approach in designing combinatorial or medicinal chemistry libraries for drug discovery. 1. A qualitative and quantitative characterization of known drug databases. *J Comb Chem* **1999**, *1*, 55-68, doi:10.1021/cc9800071.
5. Veber, D.F.; Johnson, S.R.; Cheng, H.Y.; Smith, B.R.; Ward, K.W.; Kopple, K.D. Molecular properties that influence the oral bioavailability of drug candidates. *J Med Chem* **2002**, *45*, 2615-2623, doi:10.1021/jm020017n.
6. Wildman, S.A.; Crippen, G.M. Prediction of Physicochemical Parameters by Atomic Contributions. *J Chem Inf Comput Sci* **1999**, *39*, 868–873, doi:10.1021/ci990307l.
